# Elicitors and co-factors in food-induced anaphylaxis in adults

**DOI:** 10.1186/2045-7022-3-38

**Published:** 2013-11-21

**Authors:** Stephanie Hompes, Sabine Dölle, Josefine Grünhagen, Linus Grabenhenrich, Margitta Worm

**Affiliations:** 1 Department of Dermatology and Allergology, Allergy-Center-Charité, Charité - Universitätsmedizin Charitéplatz 1, 10117 Berlin, Germany; 2 Institute for Social Medicine, Epidemiology and Health Economics, Charité Universitätsmedizin, Berlin, Germany

**Keywords:** Anaphylaxis, Co-factors, Food allergens, Celery, Wheat

## Abstract

Food-induced anaphylaxis (FIA) in adults is often insufficiently diagnosed. One reason is related to the presence of co-factors like exercise, alcohol, additives and non-steroidal anti-inflammatory drugs. The objective of this analysis was to retrospectively investigate the role of co-factors in patients with FIA. 93 adult patients with suspected FIA underwent double-blind, placebo-controlled food challenges with suspected allergens and co-factors.

The elicitors of anaphylaxis were identified in 44/93 patients. 27 patients reacted to food allergens upon challenge, 15 patients reacted only when a co-factor was co-exposed with the allergen. The most common identified allergens were celery (n = 7), soy, wheat (n = 4 each) and lupine (n = 3). Among the co-factors food additives (n = 8) and physical exercise (n = 6) were most frequent. In 10 patients more than one co-factor and/or more than one food allergen was necessary to elicit a positive reaction.

The implementation of co-factors into the challenge protocol increases the identification rate of elicitors in adult food anaphylactic patients.

## 

Anaphylaxis is a severe systemic hypersensitivity reaction [1]. The most common triggers of food-induced anaphylaxis (FIA) are tree nuts, peanuts, fish, crustacean, cow´s milk and hen´s egg [2]. Co-factors may contribute to the elicitation of a severe allergic reaction [2,3]. Therefore these factors should be considered in the diagnostic work-up of oral food challenges. Reported co-factors are e.g. physical exercise, drugs, infections, psychological stress, alcohol but also food additives [2-4].

Herein we report on large series of FIA patients who underwent double-blind, placebo-controlled food challenges (DBPCFC) to identify the eliciting allergen and relevant co-factors as part of routine clinical care.

## Findings

### Methods

We retrospectively assessed data from patients with suspected FIA who presented at the Allergy-Centre-Charité between 2007–2011. The inclusion criteria for this analysis were the onset of at least one severe pulmonary and/or one severe cardiovascular symptom in combination with the onset of gastrointestinal and/or skin symptoms. All patients were subjected to skin prick test (SPT) with commercial allergen extracts and with native materials (prick to prick) as described [5]. If suspected food allergens were not covered by the standard panel, additional SPTs were performed. Total immunoglobulin E (tIgE), specific immunoglobulin E (sIgE) and tryptase were determined with ImmunoCAP System Thermo Fisher Scientific (Uppsala, Sweden).

The DBPCFC were given titrated in five steps increasing 10-fold from 0.01 g to the total (cumulative) dose. The cumulative dose of fruits was 50 g, of tree nuts and peanuts 10 g, of soy and cow´s milk 200 ml and of cereals 100 g. The time interval between the first and the second dose was 15 min, between the following doses 30 min each. The food allergens used in the provocation tests were blinded. The basis ingredient of all meals was a hypoallergenic infant formula (Nestlé Nutrition GmbH, Frankfurt am Main, Germany). Blinding was achieved with several ingredients like peppermint-syrup (Monin, Bourges, Frankreich) cocoa powder (Krüger GmbH, Bergisch-Gladbach, Germany), orange-flavour or blackcurrant-flavour (SHS Gesellschaft für klinische Ernährung mbH, Heilbronn, Germany), oat- or rice-flakes (Demeter, Bauckhof, Darmstadt, Germany). The relation of verum and placebo challenges was 1:1. It was paused two hours between the different food challenges (whether placebo or verum).

If the suspected food allergen elicited no allergic reaction upon challenge and if the patient’s history indicated a possible role of co-factors, these were integrated into the DBPCFC. Co-factors were applied septely before the combination with the suspected food allergen. Exercise was applied 60 min after food intake. The length and intensity of physical activity was based on patient’s history and reached from 15 min to 60 min on a treadmill. Acetylsalicylic acid (ASA) was applied 60 min before food intake and doses were given according to patient´s history reaching from 100 to 500 mg. Alcohol was applied 10 min before and the food additives 30 min before the sequential food challenge. The food-additives were challenged all together with a capsule exposure including coloring agents (E 110, 122, 124, 151, 104, 127, 131, 132, 172, 120, 5 mg each; E102, 50 mg), preservatives (E 200, 211, 214, 1000 mg each; E223, 251, 100 mg each), antioxidants (E320, 321, 310, 306–309, 50 mg each), taste enhancer (E621, 500 mg), naturally occurring substances (salicylic acid, 100 mg). The placebo capsules were filled with mannit and silicium dioxide. Data processing and analysis was done with SPSS 19.0 (SPSS Inc., Chicago, IL, USA).

### Results

**Figure 1:**
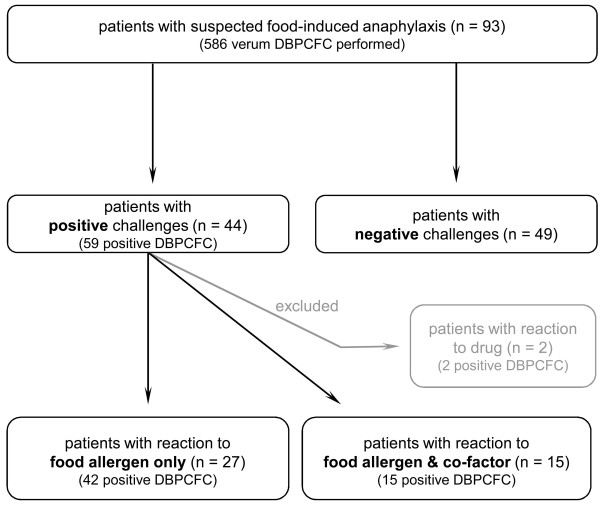
**Outcome of the double-blind, placebo-controlled food challenge (DBPCFC) in 93 patients.** Two patients reacted to the single co-factor (diclofenac and pantozol) and were excluded for further analysis.

Ninety three patients with the diagnosis of suspected FIA were included into this analysis (32 male, median age: 42 years) (Figure 1). 44 patients had positive challenge tests. 27 patients developed symptoms after the food allergen challenge and 15 reacted only after combining the food allergen with co-factors. Two patients reacted to the suspected co-factors only (diclofenac and pantozol) (Figure 1). Upon challenge most patients developed only cutaneous and/or gastrointestinal symptoms (67%) whereas in real life (upon history) reactions were more severe with 100% of patients showing respiratory or/and cardiovascular symptoms.

The most common food allergen was celery (n = 7) followed by wheat, soy (n = 4 each) and lupine (n = 3), (Table 1). These food allergens were also the most common suspected elicitors of anaphylaxis (wheat in 41 patients, celery in 40, soy in 21 and lupine in 14 patients). In 5 patients the combination of one co-factor with a single food allergen induced an allergic reaction (Table 2). In 7 patients more than one co-factor or more than one food allergen was required to induce a positive reaction. Three patients required the presence of 2 or 3 co-factors in combination with 2 or 3 food allergens to induce the reaction. Food additives (n = 8), physical exercise (n = 6), ASA (n = 4), alcohol (n = 3) and clindamycin (n = 1) were identified as co-factors. Among food allergens in combination with co-factors, wheat (n = 10) most frequently induced anaphylaxis followed by celery (n = 6), seafood (n = 3) and hazelnut (n = 2).

**Table 1:** Suspected and proven single food allergens (182 challenges)

	**Elicitors**	
	**Suspected**	**Confirmed**
**Food**	**n**	**n**
**Vegetables**	**44**	**10**
Celery	40	7
Bell pepper	2	1
Broccoli	1	1
Potato	1	1
**Legumes**	**46**	**8**
Soy	21	4
Lupine	14	3
Peanut	11	1
**Cereals**	**43**	**6**
Wheat	41	4
Buckwheat	1	1
Spelt	1	1
**Fruits**	**7**	**5**
Apple	3	1
Pear	2	2
Nectarine	1	1
Orange	1	1
**Animal derived food**	**19**	**5**
Shrimps	9	1
Cow´s milk	4	1
Salmon	4	1
Hen´s egg	2	2
**Spices & others**	**12**	**4**
Curry	8	2
Sesame	3	1
Horseradish	1	1
**Tree Nuts**	**20**	**3**
Hazelnut	16	2
Almond	4	1
**Others**		
Cereal bar	1	1

**Table 2:** Patients characteristics and challenge details of positive DBPCFC (n =15) upon inclusion of co-factors

			**Co-factor**						**Food**					
**Sex**	**Age (in years)**	**No. of cofactors**	**Additives**	**Exercise**	**ASA**	**Alcohol**	**Clindamycin**	**No. of foods**	**Wheat**	**Celery**	**Seafood**	**Hazelnut**	**Sesame**	**Tomato**
M	24	**1**		x				**1**	x					
F	26	**1**	x					**1**		x				
F	66	**1**			x			**1**		x				
M	51	**1**			x			**1**	x					
M	51	**1**		x				**1**	x					
F	35	**1**	x					**2**		x		x		
M	54	**1**	x					**2**	x	x				
F	63	**1**	x					**2**	x	x				
F	29	**1**				x		**4**	x	x		x	x	
M	40	**2**	x	x				**1**	x					
F	51	**2**	x		x			**1**			x			
F	28	**2**		x			x	**1**	x					
F	33	**2**			x	x		**1**			x			
F	34	**2**	x	x				**2**	x					x
M	52	**3**	x	x		x		**2**	x		x			
		Sum of co-factors:	8	6	4	3	1	Sum of foods:	10	6	3	2	1	1

(M = male, F = female; ASA = acetylsalicylic acid).

**Figure 2:**
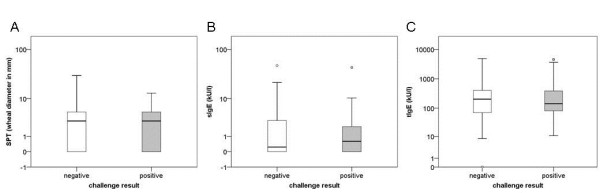
**SPT-, sIgE- and tIgE-results in relation to positive (n = 59) and negative (n = 527) challenge results.** Median (min – max) of **A)** SPT-values in the positive challenge group: 3 (0–13) mm and negative challenge group: 3 (0–30) mm; **B)** sIgE-values in the positive challenge group: 0.60 (0.00-43.7) kU/l and negative challenge group: 0.24 (0.00-47.7) kU/l; **C)** t-IgE-values in the positive challenge group: 142 (11–4538) kU/l and negative challenge group: 203 (0–4887) kU/l.

The values of SPT, sIgE and total IgE were distributed similar between positive and negative challenge results (Figure 2). In 4 patients tryptase (>11.5 μg/l) were elevated, but below 20 μg/l, an underlying mastocytosis was excluded by a thorough inspection of the skin.

### Discussion

In 16% of patients with anaphylaxis the co-exposure of co-factors with food-allergens was necessary to elicit a reaction. The underlying pathomechanism of co-factors in FIA is not known, but an impairment of the gastroduodenal permeability leading to an increased absorption of allergens has been discussed [6].

The finding that in real life reactions were more severe than upon challenge can be explained due to the fact that for safety reasons the challenge meals were given titrated and the supervised food challenges were stopped immediately when objective symptoms occurred.

The most common single food allergen causing anaphylaxis was celery, which is in line with previous data [7]. Celery has been reported in conjunction with co-factors to induce anaphylaxis previously [7,8]. However, the most frequent reported food allergen in combination with co-factors is wheat and wheat-dependent exercise-induced anaphylaxis is well known. Nevertheless other food allergens e.g. tree nuts have been reported to induce allergic reactions in combination with physical exercise as well [6]. Why exercise is a frequent co-factor in wheat allergy is not known. An increased stability of wheat allergens to gastrointestinal enzymes resulting in an increased absorption upon physical activity may be relevant [9].

Some patients reacted only when multiple co-factors and several food allergens were present. This observation has only been described in single case-reports previously. Aihara et al. reported the requirement of the simultaneous intake of two food allergens to provoke food-dependent exercise-induced anaphylaxis [10]. The additive effect of co-factors has been described for exercise together with ASA as well [6] and we reported previously a case, where wheat, exercise, alcohol and food additives were necessary to elicit the reaction [4]. Despite the complex procedure to evaluate co-factors in clinical practice, their role should be analysed in prospective controlled clinical trials to unravel how frequent they are in unselected patient populations.

DBPCFC can be performed in patients with FIA, although there is a potential risk for severe reactions. In 47% of the patients the cause and circumstances to elicit their reaction were identified. Considering co-factors for the challenge protocol and including them in provocation tests with previously negative results we increased the rate of positive reactions. Although, the underlying mechanism is not yet understood, the implementation of co-factors into current challenge protocols seem to be worthwhile to improve the identification rate of elicitors in adult patients with FIA.

## Abbreviations

DBPCFC: Double-blind placebo-controlled food challenges; FIA: Food-induced anaphylaxis; SPT: Skin prick test; tIgE: total immunoglobulin E; sIgE: Specific immunoglobulin E; ASA: Acetylsalicylic acid.

## Competing interest

All authors declare that they have no competing interests.

## Authors’ contributions

SH analyzed the data, created the figures and wrote the first draft of the manuscript. JG and SD supported data acquisition and analysis. LG supported data analysis. MW developed the concept and analysis of the data, performed data interpretation and wrote and revised the article. All authors have approved the final version of the manuscript.
